# Cases Illustrative of the Different Forms of Phagedenic Ulcer

**Published:** 1826-09

**Authors:** G. Babington

**Affiliations:** Surgeon to the Lock Hospital.


### PHAGEDENIC ULCERS.

*Cases illustrative of the different Forms of Phagedenic Ulcer.* Communicated by G. Babington, Esq. Surgeon to the Lock Hospital.

### I. *Case of Phagedenic Ulcer, treated with Mercury.*

Ann Collins, ætatis twenty-one, was admitted into the Lock Hospital, on the 29th of January, 1825, with a sloughing sore, which had destroyed the extremity of the urethra, and extended upwards as far as the clitoris. It was spreading rapidly in every direction. The surface of the sore was dark, and the surrounding parts were much tumefied, and of a bright florid red colour. It appeared by her description, that a small sore was first discovered near the orifice of the urethra, a fortnight before her admission. In a few days it became exceedingly painful, and rapidly increased in size. The pain was extremely severe and constant, but was much augmented on passing the urine, so that she had voided none for more than twenty-four hours previous to her admission into the hospital.

She was ordered to take five grains of the Pil. Hydrarg. twice in the day; to keep an aqueous solution of Opium, covered with a bread-poultice, as a constant application to the sore; and to fumigate it every night and morning with a drachm of Cinnabar.

Jan. 31st.—The pain was in no degree relieved; nor was the progress of the sore arrested.

Directed to continue the Pil. Hydrarg. and the same local treatment, and to take one grain of Opium every four hours.

February 1st.—The opium relieved the pain, but there was as yet no change in the appearance of the sore. She complained that she suffered from the fumigation, which she was directed to discontinue.

The quantity of Opium was diminished, so as to be taken only every eight hours.

3d.—To-day the gums showed signs of tenderness. There was a manifest improvement in the appearance of the sore, which was quite free from pain. The surrounding parts were less inflamed; the nymphæ less tumefied, and the colour less florid; the surface of the sore also was cleaner.

The Opium to be taken only twice in the day; the other remedies to be continued.

5th.—The surface of the sore was clean; the tumefaction and inflammation of the surrounding parts was nearly gone. The affection of the gums continues, but is moderate in degree.

Directed to omit the Opium altogether.

8th.—The solution of opium and the poultice were changed for a wash, consisting of a solution of the nitrate of mercury in lime-water. The progress of the sore to cicatrisation was regular, though not rapid. The mercurial action could not be kept up in the degree that was desirable, in consequence of the supervention of ulceration round one of the dentes sapientiæ, which had scarcely protruded from the gum. It was necessary to compensate the deficiency of effect by a prolongation of the course.

At the end of the month the sore was healed, and the Pilula Hydrargyri was continued as before, twice in the day, till the 14th of March; when, being perfectly well in all respects, she left the hospital.

II. *Case of Phagedenic Ulcer, treated with Opium, both locally and generally.*

Sarah Starkey, ætatis twenty, was admitted into the Lock Hospital, on the 21st April, 1825, with a large foul sore, situated on the inside of the right thigh, not far from the pudenda. It was larger than a cleft orange, very dark and foul on the surface, and surrounded by an inflamed border of a dark red colour; the edges were tumid, and partially everted. Its progress was attended with very severe and constant pain, which almost entirely prevented sleep at night. The ulcer appeared, by her description, to have originated, nearly three weeks before her admission, in what she supposed to be a common pimple. Its increase had latterly been very rapid, and the pain had been proportioned to the violence of its progress. She had also discharge, and a small sore on the labium pudendi.

She was directed to take one grain of Opium every four hours, and to apply to the sore lint dipped in an aqueous solution of Opium, which was covered by a common bread-and-water poultice.

22d.—The pain was subdued. She had obtained some sleep during the night.

23d.—She continued free from pain, and the aspect of the sore began to improve: the edges were less swollen, the progress appeared to be arrested, and the slough began to separate from the surface.

Ordered to take the Opium only once in six hours, and to continue the applications.

26th.—The surface of the sore was covered with healthy granulations, and the edges were disposed to cicatrise.

The Opium was directed to be taken only three times in the day.

28th.—As the sore continued to improve daily, and was considerably diminished in size, the Opium was administered only twice in the day.

29th.—The pain returned in the evening, and the sore again assumed a dark unhealthy appearance, which threatened a return of the sloughing.

To take the Opium every six hours.

May 5th.—The sloughing was soon arrested by the Opium, but the sore was languid : a thin layer of slough remained on it, which separated very slowly; and the granulations of those parts from which it had separated were very slow in their growth, and were neither florid nor vigorous.

She was ordered to continue the Opium, and to take, in addition, a pint of the Lisbon diet-drink daily.

12th.—A violent diarrhœa came on, which was supposed to be principally owing to the long administration of large doses of Opium. The Opium was accordingly omitted, and a small quantity of Tincture of Ginger was added to the Sarsaparilla. These changes answered the object, and all disorder of the bowels had ceased on the 14th; but the pain in the sore had returned with as much violence as ever, and it began again to slough with great rapidity. As it was evident that opiates were necessary, and the stomach and bowels would not bear the extract, the Tinct. Opii was substituted for it, in the dose of twelve minims every six hours. Under its use, the disorder of the bowels did not recur; the pain soon ceased; the sore again assumed a healthy aspect; and, as the state of the constitution improved under the Sarsaparilla, the granulations acquired vigour, and the cicatrisation proceeded regularly. At the end of the month, the ulcer was reduced to the size of a shilling, but, from the great destruction of skin in the different attacks of sloughing, the progress was latterly more tardy, and it was not until the 21st of June that it was entirely healed.

*Case of Phagedenic Ulcer, treated principally with Cinchona and Sulphate of Quina.*

John Bourke, ætatis twenty-four, was admitted into the Lock Hospital, on the 14th of January, 1826, with an extensive ulcer in the right groin, formed by a bubo which had burst about a month before. The surface was unhealthy, and the edges were becoming jagged and irregular. There was another ill-conditioned sore on the scrotum, and also a primary sore on the glans penis. He appeared to have passed through a course of mercury before his admission; but, as the effect had been violent, it was uncertain whether it had been conducted with sufficient regularity. However this might be, the appearance of the sores, and the state of the general health, forbade the administration of mercury, at least for the present.

He was accordingly directed to take a pint of Decoction of Sarsaparilla daily, and to apply a Linseed Poultice to the ulcers.

On the 21st, the sores showed some disposition to spread. The tongue became furred, the pulse hurried, the skin hot, and the whole system greatly disordered.

He was directed to discontinue the Sarsaparilla, and to take the Haustus Salinus, with half a drachm of the Liquor Antimonii Tartarisati, and a drachm of Sulphate of Magnesia three times a-day.

24th.—The skin cooler, and the pulse somewhat less active; but the tongue is still foul. The sores have begun to slough.

To omit the Saline Medicines, and to take the Mistura Cinchonæ, with half a drachm of the Sulphate of Magnesia, three times in the day.—To apply to the ulcers Treacle spread on lint, and covered with a Bread Poultice.

28th.—The sores are rapidly extending by sloughing, especially that on the side of the scrotum. The tongue is not less furred, and is assuming a darker appearance. He is very restless, and gets no sleep, but does not complain of much pain in the sores.

To continue his medicines.

31st.—Both sores have been sloughing rapidly, and within the last few days they have extended to more than double their former size. There is little pain; the edge is not tumid or everted, but has a narrow border of yellow slough, surrounded by a circle of very pale-coloured inflammation, extending to the distance of little more than half an inch. The slough does not separate in a distinct mass, but gradually disappears as a fresh portion of skin is involved. It is as if the slough melted down into pus on the side of the sore, while it spread on the side of the sound skin.—This morning there has been hemorrhage from the sore in the groin, to a very considerable extent: which is not, however, sufficiently deep to have reached the larger blood-vessels. The pulse is extremely depressed; the countenance pale; the tongue is becoming dark and dry.

Ordered to take an ounce and a half of the Mist. Cinchonæ, with a drachm of Tincture of Ginger, every six hours; and to apply to the sore lint dipped in a solution of Muriatic Acid, in the proportion of half a drachm of the acid to half a pint of water, covering the whole with a Linseed Poultice.—To take also at night fifteen grains of Dover's Powder, in Camphor Mixture.

February 3d.—The hemorrhage has not returned, but the sloughing continues. The bottom of the sore on the thigh looks cleaner as the cellular membrane is destroyed, and the muscles are exposed; but the edge spreads as before. The two sores have been broken into one, by the destruction of the skin which separated them. The tongue is still black, and the pulse continues much depressed.

Sulph. Quininæ gr. ij.; Ext. Gentian. q. s. tertiâ quaque horâ cum haustu sequenti.—Infusi Rosæ ℥ iss.; Acid. Sulph. dil. m. xij. M. Potassæ Supertart. ʒ ij. omni mane.

11th.—The sores have been spreading less rapidly; the tongue begins to assume a better appearance, and the appetite improves; but the pulse is still very weak.

18th.—The slough has now separated; the edges of the sores begin to show more activity, and to close in; the surface is red, but granulates slowly. The pulse still weak; the tongue quite clean, and the appetite good.

April 8th.—The sores have continued to heal, though slowly, from the great destruction of skin. The health is restored, the appetite good, and he gains flesh.

Omittatur Quinina.

The subsequent treatment offered nothing remarkable. The sores healed regularly, but were not entirely closed until the 21st of May.

---

